# Enhancing the natural absorbing capacity of rivers to restore their resilience

**DOI:** 10.1093/biosci/biae090

**Published:** 2024-09-19

**Authors:** Ellen Wohl, Kirstie Fryirs, Robert C Grabowski, Ryan R Morrison, David Sear

**Affiliations:** Department of Geosciences at Colorado State University, in Fort Collins, Colorado, United States; School of Natural Sciences at Macquarie University, North Ryde, New South Wales, Australia; Centre for Water, Environment, and Development at Cranfield University, Cranfield, England, United Kingdom; Department of Civil and Environmental Engineering, Colorado State University, Fort Collins, Colorado, United States; Department of Geography and Environmental Science, University of Southampton, Southampton, England, United Kingdom

## Abstract

Resilience, which can also be described as absorbing capacity, describes the amount of change that a system can undergo in response to disturbance and maintain a characteristic, self-sustaining regime of functions, processes, or sets of feedback loops. Rivers exhibit varying levels of resilience, but the net effect of industrialized anthropogenic alteration has been to suppress river resilience. As changing climate alters the inputs to rivers and human modification alters the morphology and connectivity of rivers, restoration increasingly considers how to enhance resilience. Characteristics that underpin river absorbing capacity include natural regimes, connectivity, physical and ecological integrity, and heterogeneity. River management emphasizing channel stabilization and homogenization has reduced river absorbing capacity. We propose that the paths to restoring rivers include defining relevant measures of absorbing capacity and understanding the scales of restoration and the sociopolitical elements of river restoration. We provide a conceptual framing for choosing measures that could be used to assess river absorbing capacity.

Many rivers of the world are experiencing exacerbated disturbances driven by climate change, such as increases in flooding, wildfires, and droughts, as well as warming river temperatures, with some of these extreme events being the most severe and intense in modern history (e.g., Liu et al. 2018, Swain et al. 2020). These disturbances interact with increasing human consumptive demands for freshwater and other riverine resources (Best 2019) and human-induced pollution and contamination. Adjustments to aquatic ecosystems and their underlying physical structure and function resulting from changes in the frequency, magnitude, intensity, and timing of extreme events are challenging the preconceived notions of stable river systems held by many communities (Hooke 2015). Faced with such challenges, river management and government agencies are increasingly turning toward approaches based on the concept of resilience.

*Resilience* has been defined in the literature to accommodate numerous disciplines (table 1). We define it as the amount of change that a system can undergo while maintaining a characteristic and self-sustaining regime of processes, functions, and sets of feedback loops that sustain or improve ecosystem health (Walker and Salt 2006, Thoms et al. 2018). River resilience tends to occur as the river adjusts one or more of the following capacities: absorbing capacity that allows the recovery of a system without causing irreversible or wholesale change to forms and functions, with the river being able to absorb any changes to internal inputs and external drivers that affect the system; adaptive capacity that describes the capacity of social–ecological systems to adjust to potential damage and take opportunities to respond to consequences (e.g., Folke 2006, Folke et al. 2010); and transformative capacity that allows development or creation of a new system (Thoms et al. 2024). In this article, we are focusing on the restoration of the absorbing capacity of rivers, rather than the broader concept of resilience.

**Table 1.** Definitions of resilience and related terms.

**Term**	**Definition and context**	**Representative references**
*Resilience*	The amount of change that a system can undergo and remain within the same regime that retains similar function, structure, and feedback loops. Resilience is measured in terms of response versus disturbance and includes indicators of early warning signals of tipping points (e.g., critical transitions, or flickering).	Holling 1973, Walker et al. 2004, Walker and Salt 2006, Phillips and Van Dyke 2016, Thoms et al. 2018, Moore and Schindler 2022, Scheffer et al. 2015
	Designed resilience used in engineering when disturbances and shocks are known and the system is designed and built to withstand these known shocks: engineering resilience focuses on resistance to disturbance, describing a system near an equilibrium steady state	Colding and Barthel 2019, Fuller et al. 2019
	The rate at which a system returns to its equilibrium, often measured as its reciprocal, the return time for the disturbance to decay to some specific fraction of its initial value; shorter return times equate to more resilience	Donohue et al. 2016, Hillebrand et al. 2018
	The ability of a socioecological system to adapt and change in response to slow or fast perturbations in the ecological or social systems involved through positive and negative feedback loops. Socioecological systems are complex and adaptive as a result of self-organization and nonlinear dynamical behavior; therefore, they are characterized by change, not stability.	Holling and Gunderson 2002, Biggs et al. 2015, Parsons and Thoms 2018
Related terms	Disturbance: any relatively discrete event in time that disrupts ecosystem, community, or population structure and changes resources, substrate availability, or the physical environment	White and Pickett 1985
	Disturbance regime: the spatial pattern and statistical distribution of events in terms of frequency, magnitude, and duration of associated changes in the physical environment	Montgomery 1999
	Sensitivity: how much the river corridor responds to an increment of change	Wade et al. 2017

Our system of interest is the river corridor, which includes the active channels, adjacent floodplain, and subsurface hyporheic zone (figure 1; Newson 1992, Harvey and Gooseff 2015). Our focus is on physical processes, such as the flows of water, sediment, and organic matter (e.g., wood), which are fundamental to river functioning and the creation, maintenance, and turnover of ecological habitats. Referring to a river corridor rather than just a river channel explicitly recognizes the three-dimensional connectivity and interaction among these components. Any given length of river corridor exists within a river catchment and network, which includes the entire system of river corridors draining to a specified point in the landscape. The disturbances against which we assess river corridor absorbing capacity can be natural, such as flood or drought, or anthropogenic, such as flow regulation, channelization, and changes in land cover (Fuller et al. 2019). Disturbances can be direct and on site or indirect and off site. Disturbances can also be discrete, relatively short events, such as a flood, or slow phenomena, such as climate change, that persist and perhaps increase in magnitude with time (Glasby and Underwood 1996). Both fast and sustained disturbances commonly interact and can occur coevally.

**Figure 1.**
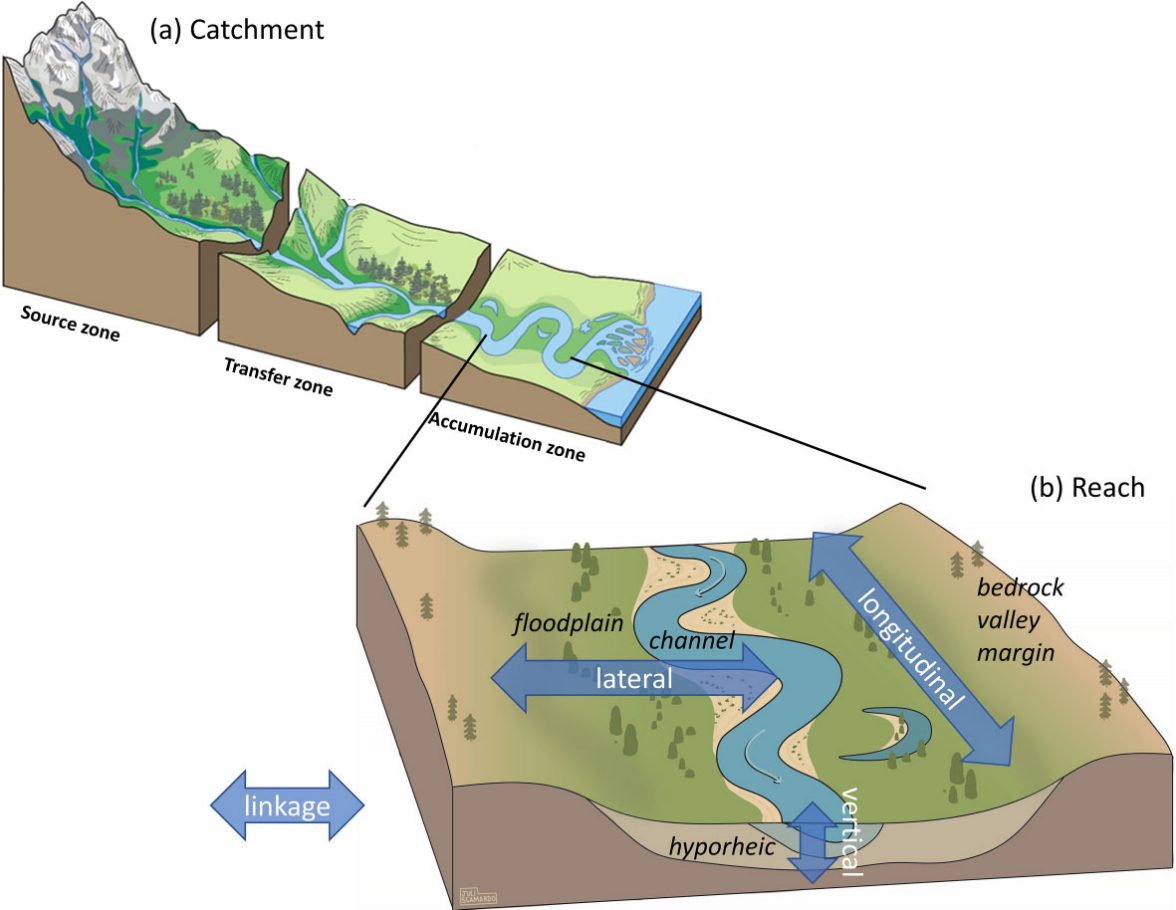
Schematic illustration of a reach-scale river corridor, including the active channels, floodplain, and underlying hyporheic zone. The translucent arrows indicate three primary dimensions of connectivity within the river corridor: longitudinal (upstream–downstream), lateral (channel–floodplain), and vertical (surface–subsurface). The reach exists within the spatial context of a river catchment. Source: Catchment-scale illustration (a) modified from Thornberry-Ehrlich, US National Park Service, https://www.nps.gov/subjects/geology/fluvial-landforms.htm. Reach-scale block diagram (b) courtesy of Julianne Scamardo.

The ideas of resilience and absorbing capacity are attractive to the broader community and policymakers. However, misunderstanding and miscommunication of what river resilience means is creating a misleading sense of stability, certainty, and security where these characteristics cannot be realistically delivered (Gould and Lewis 2021). In societal and engineering contexts, managing systems for resilience has traditionally been focused on creating stability by repelling or resisting changes from a disturbance to protect communities and infrastructure (Holling and Meffe 1996, Rahel 2022). This attempt at top-down control has produced a false perception that such events can be managed. As a result, the concept of resilience has been misinterpreted and misused within the fields of river engineering and restoration.

In the context of river corridors, the key to understanding absorbing capacity is understanding how river ecosystem forms and functions vary across space and time. Approaches to enhancing absorbing capacity in a river restoration context include disconnecting intensive land uses that alter inputs to the river network and restoring natural levels of connectivity within the river corridor. Disconnecting intensive land uses can involve activities such as restoring riparian vegetation buffers or floodplain wetlands or removing or plugging floodplain tile drains or ditch drains. The restoration of natural connectivity involves methods such as removing barriers to channel lateral migration, including artificial levees and bank stabilization, providing adequate room for the river to adjust, and restoring naturally occurring limits on longitudinal connectivity, such as logjams and (in the northern hemisphere) beaver (*Castor* spp.) dams.

We argue that river restoration efforts must be conducted with building or rebuilding the river corridor’s absorbing capacity in mind. In this article, we begin by introducing the concept of a river corridor and define the properties of its absorbing capacity. We then describe conventional restoration initiatives and contrast them with alternative approaches that could be used to enhance the absorbing capacity of rivers. We finish by suggesting some metrics for assessing river absorbing capacity that could be used to evaluate the success or otherwise of building or rebuilding resilience via river restoration.

## River corridors in the context of absorbing capacity

Because river corridors exist within catchments, the state of the river corridor reflects catchment processes that create fluxes of water, solutes, sediment, and organic matter throughout a river network. These catchment processes drive the physical creation, maintenance, and turnover of habitats within the river corridor and influence water chemistry, nutrient cycling, and dispersal to affect ecological communities (Poole 2002, Pelletier et al. 2020).

River corridors and ecosystems also exist within a societal context (Corenblit et al. 2024, Thoms et al. 2024). Assessing river corridor absorbing capacity requires attention to attributes that confer this capacity in river corridors (Hodgson et al. 2015, Parsons et al. 2016, Gould and Lewis 2021). These include biophysical characteristics such as spatial freedom for rivers to laterally adjust or to mitigate floods (Biron et al. 2014, Klijn et al. 2018), but they also include socioeconomic influences such as nature-based restoration (Norman et al. 2022) and conceptualizations for water governance (Martin and Holley 2024).

Fundamental to assessment of absorbing capacity is the clear definition of systems, which, at any place and time, is an expression of past evolution, contemporary processes, and the timing and sequencing of disturbances that occur in the surrounding catchment and river network (Gregory and Walling 1976, Schumm 1977, Sear 1994, Brierley and Fryirs 2005). River absorbing capacity should therefore be considered a trait of any given river corridor ecosystem relative to its expected degree of adjustment or change. However, given that current and future climate or land use conditions may be outside those that have been experienced by river systems, *expected* is an important term to define. In the context of river restoration, expected change needs to be considered over appropriate spatial (1 kilometer to 1000 square kilometers) and temporal (e.g., multiple decades) scales. A reach of river corridor exists within a larger catchment, and decades or longer can pass before a river fully adjusts to natural or anthropogenic disturbance.

When an external disturbance is too large to be absorbed by a system and overcomes the system's absorbing capacity, the system can be pushed over a tipping point into a new system state of different forms or functions (Lenton 2013, Delong et al. 2024). Tipping points may depend on the presence of strong positive feedback loops that amplify responses to a disturbance. For example, climate change is inducing longer-term changes in flow regimes as glaciers melt and rainfall replaces snow and short-term disturbances as rainfall intensity increases (Gudmundsson et al. 2021). These changes can then alter water, nutrient, and sediment inputs to river networks and fluxes through river corridors, which, in turn, alter river corridor forms and functions (East and Sankey 2020). A new ecological state with a different community structure and function may be produced through the direct impacts of these physicochemical changes on individuals and changes in habitat availability, suitability, or connectivity, which affect population size or occupancy (Chase et al. 2020, Pelletier et al. 2020).

In river corridors, examples of tipping points that enhance absorbing capacity might include the reintroduction of beaver (where appropriate), which are natural ecosystem engineers that rapidly transform river corridors by modifying connectivity and restructuring ecological communities. Beaver modifications create positive feedback loops resulting in benefits to society, including reduced flood levels downstream, biodiversity gain, water quality improvement, and carbon sequestration (Puttock et al. 2017, Laurel and Wohl 2019, Wohl 2021).

In river corridors with histories of modification stretching back hundreds or thousands of years, engineered states have been maintained by modified disturbance regimes (e.g., flow regulation) and management paradigms (Brown et al. 2018). For example, in some places where river corridors with multiple channels would be expected, human activities have simplified rivers to a single, stabilized channel (Brown et al. 2018), either as a result of changes in upland cover and sediment yield that cause floodplain alluviation and development of an incised channel (Trimble 2013) or as a result of channel engineering (Pišút 2002, Walter and Merritts 2008). Ongoing human preference for large, single, straight river channels requires persistent maintenance (Moore and Rutherfurd 2017), rather than allowing the river to readopt its expected multichannel form. Such engineered states may become more sensitive to rapid or unexpected shifts in the event of a disturbance because of loss of absorbing capacity. In these scenarios, river restoration can represent an intentional disturbance designed to achieve an increase in river corridor absorbing capacity, leading to a system state that is capable of persistence and functional diversity in response to future disturbances. In effect, process-based restoration can be seen as an example of a positive (societal) tipping point (Lenton et al. 2022).

## Characteristics that create and sustain river corridor absorbing capacity

River corridors are complex, dynamic, and evolving systems that are characterized by adjustment and change, not stasis. The dynamism of river corridors allows them to adapt, self-organize, and exhibit nonlinear and emergent dynamical behavior when disturbed (Phillips and Van Dyke 2016). A river corridor will change over time, but the key to understanding absorbing capacity is how river forms and functions vary across spatial and temporal scales and the effects on ecological communities (Death 2024).

**Figure 2.**
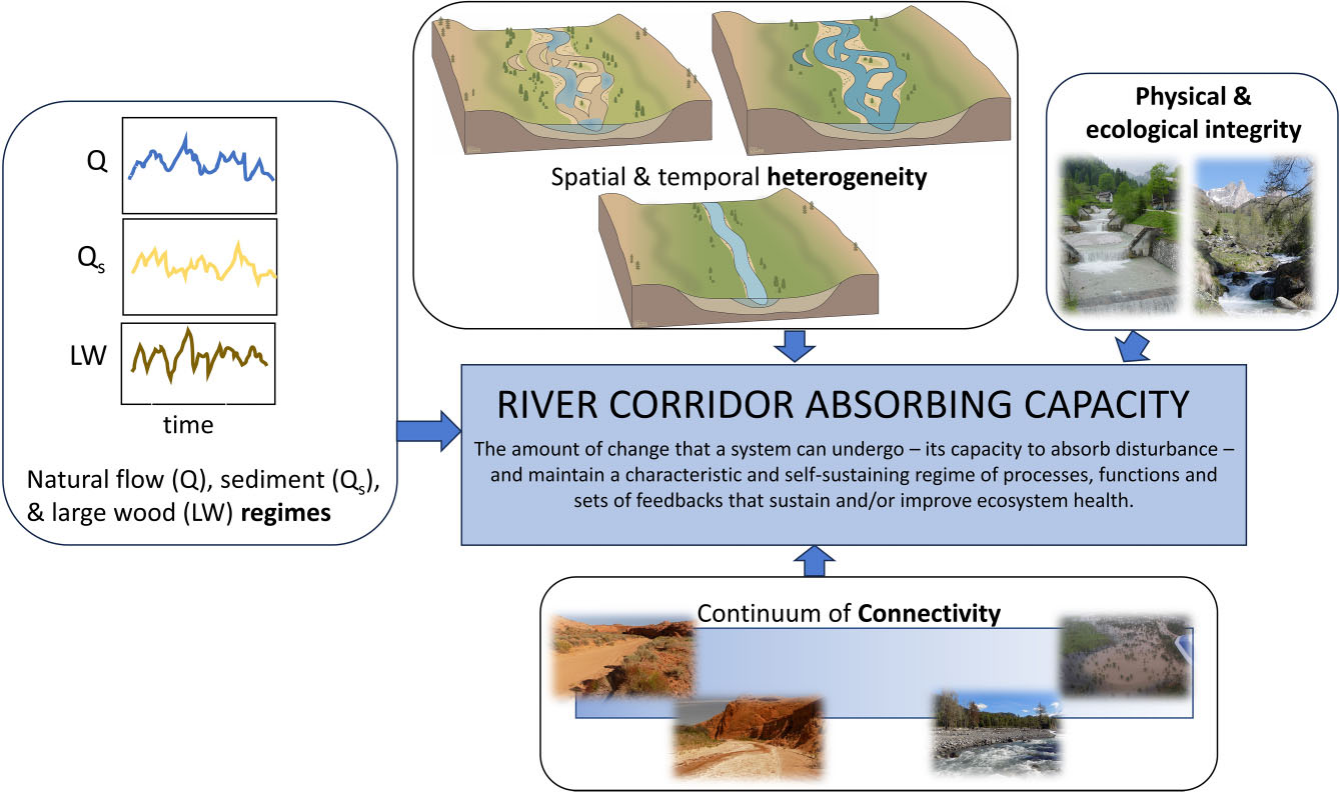
Schematic illustration of forms, process, and functions that contribute to the absorbing capacity of a river corridor.

River corridor absorbing capacity is created and sustained when geomorphic form and process and ecosystem structure and function are occurring and operating as expected for any given reach, river network, or catchment, across space and through time (Montgomery 1999, McCluney et al. 2014, Fryirs 2017, Wohl et al. 2022). Forms, processes, and functions that contribute to absorbing capacity are illustrated in figure 2.

### Natural regimes

Underpinning contemporary understanding of rivers as dynamic entities is the idea of natural regimes (figure 2). *Regime*, in the present article, refers to temporal variability in magnitude, frequency, duration, and timing of a flux, whether the flux is water, sediment, or another material. The physical, biological, and chemical characteristics of river ecosystems reflect regimes of water (Poff et al. 1997), sediment (Wohl et al. 2015a), large wood in forested environments (Wohl et al. 2019b), and solutes and particulate organic material (Tank et al. 2010, Bernhardt et al. 2017). Natural river regimes also include biological components and, therefore, include fluxes of biota and their different life stages (e.g., dispersal and migration).

Relatively natural regimes and the associated disturbances characterize a river corridor with absorbing capacity in which physical forms and biotic communities are adjusted to these regimes (Timpane-Padgham et al. 2017). In contrast, a river corridor with altered flows downstream from a dam, for example, can lack absorbing capacity because channel form has adjusted to elimination of peak flows and an unchanging base flow, thermal diversity required to support diverse biotic communities has declined, and the habitat and flow-driven dispersal necessary to support native riverine organisms are no longer present (Beechie et al. 2006, Poff et al. 2007).

### River corridor integrity

Physical and ecological integrity promotes absorbing capacity in river corridors (figure 2). Physical integrity describes a set of active river processes and landforms wherein the river corridor maintains an expected structure and function because it is capable of freely adjusting in response to fluctuating inputs and boundary conditions (Graf 2001, Brierley et al. 2008). A river corridor that lacks physical integrity is likely to have a suppressed or compromised physical structure and function, such as that created by artificial bed and bank stabilization or levee construction or, increasingly, poorly designed restoration schemes (Doyle and Lave 2021). The ability to change through time in response to changing inputs and boundary conditions is key to physical integrity.

Ecological integrity is commonly defined as the ability of an ecosystem to support and maintain a community of organisms that has species composition, diversity, and functional organization comparable to those of natural habitats within a biome (Parrish et al. 2003). A river corridor with ecological integrity includes diverse species and age distributions within a species (as constrained by site-specific factors such as habitat availability) facilitating survival and recolonization of some individuals and species following disturbance (Timpane-Padgham et al. 2017). Biota in a river corridor that lacks ecological integrity are likely to be less diverse and less likely to find refugia and dispersal pathways (Peipoch et al. 2015).

In natural river corridors with absorbing capacity, ecological and physical integrity are codependent and interact, perhaps most easily comprehended in the action of ecosystem engineers such as vegetation (Gurnell 2014), spawning salmonids (Tiegs et al. 2009), or beaver (Curran and Cannatelli 2014). Research is revealing that this codependence operates across a range of scales. Biological processes alter bed, bank, and floodplain sediment stability, resulting in changing channel pattern (Tal and Paola 2007) and the distribution of flow energy (Tabacchi et al. 2000). These interactions are commonly underrecognized and incorporated in contemporary restoration practice. Fundamentally, physical and ecological integrity enhance absorbing capacity by facilitating the ability of the river corridor to adjust to disturbance while maintaining forms and functions.

Underpinning both physical and ecological integrity are connectivity and spatial and temporal heterogeneity of the river corridor.

### Connectivity

*Connectivity* describes the degree to which matter (water, solutes, sediment, organic matter) and organisms can move spatially across a landscape or ecosystem (figure 2; Fryirs 2013, Wohl 2017). Connectivity can be characterized in the three dimensions of longitudinal (upstream–downstream), lateral (channel–floodplain, uplands–river corridor), and vertical (surface–subsurface; figure 1; Ward 1989). Connectivity in river corridors occurs along a continuum of time and space in which different forms of matter and different organisms can experience differing degrees of connectivity (and, by implication, disconnectivity) through time (Wohl et al. 2019a). Some systems are naturally disconnected at different spatial scales and over different timeframes, whereas others are naturally connected (Fryirs et al. 2007). The degree to which a system is expected to be connected, and over what timeframe and spatial scale, influences physical response to disturbance and the manifestation of adjustment and change—and, therefore, absorbing capacity—in river corridors (Fryirs 2013). The degree of connectivity in a system also influences the biological response to disturbance by providing refugia during disturbance and allowing organism dispersal and recruitment (Timpane-Padgham et al. 2017, Van Looy et al. 2019).

Human modifications to river corridors have both increased and decreased different dimensions of connectivity, the positive or negative effects of which are contingent on the internal operations of the system affected and whether connectivity or disconnectivity is expected. For example, the construction of dams has had a negative impact in many river corridors by decreasing longitudinal connectivity, reducing physical integrity by altering downstream fluxes of material, and reducing ecological integrity by limiting migration and dispersal of native biota (Graf 1999, Merritt and Wohl 2006). Artificial levees have had a negative impact by decreasing lateral connectivity through disconnecting the active channel and the floodplain, leading to reduced physical and ecological integrity and absorbing capacity (Beechie et al. 2013, Nardi et al. 2018, Knox et al. 2022). Long histories of land drainage for intensive agriculture have negatively increased longitudinal connectivity from uplands to river corridors through the extension of the natural river network, resulting in the delivery of eroded soil and the burial of former complex floodplain wetlands (Brown et al. 2018). However, in other situations, restoration to enhance connectivity has had positive impacts on river absorbing capacity. Levees have been removed to allow floodwaters onto floodplains to restore floodplain wetlands. There are also many examples where disconnectivity has been restored as part of decreasing sediment and nutrient delivery into river networks (e.g., riparian buffers; Dunn et al. 2022), sometimes referred to as *managing-at-source* approaches to sediment oversupply (Fuller et al. 2023, Fryirs et al. 2024).

Restoring to natural levels of connectivity may not always be desirable, however, or conducive to absorbing capacity in cases where human alterations can be exacerbated by restoring natural connectivity. Invasive species that are currently limited in their range by human-built dams (Barnett and Adams 2021) or allowing highly polluted waters to access floodplain wetlands by removing artificial levees provide examples of scenarios in which trade-offs between restoration of desired processes and unintended consequences in highly altered river corridors must be considered.

Fundamentally, maintaining or restoring natural levels of connectivity can enhance the absorbing capacity of the river corridor by enhancing the ability of river corridors forms and functions to adjust to and recover from disturbance. Connectivity can also enhance the ability of riverine organisms to avoid disturbance and recolonize following disturbance.

### Spatial and temporal heterogeneity

*Spatial heterogeneity* refers to the variability of river corridor structure and the extent to which it deviates from a homogeneous, uniform configuration (figure 2; Wohl 2016). Examples include downstream or cross-channel variations in channel bed substrate, bank stratigraphy, channel cross-sectional area and symmetry, bedforms, or planform, and floodplain width, topography, stratigraphy, and hydrological connectivity with the active channels. However, this concept also recognizes that not all river corridors are naturally complex, and indeed, some are expected to be relatively simple in both their structure and function (Fryirs and Brierley 2009). Spatial heterogeneity creates a shifting diversity of habitat and resources for organisms and refugia to which flora and fauna are adapted (Poole 2002, Sear 2010, Van Looy et al. 2019). The definition of spatial scale, although commonly driven by perceived physical or ecological boundaries in the landscape that differentiate disturbance regimes and ecosystem responses, may also be defined according to biota and processes (Hawley 2018, Beechie et al. 2022).

A spatially heterogeneous river corridor includes multiple individual features that respond differently to disturbance. Some portions of the river corridor, such as densely vegetated floodplain patches or channels that branch and rejoin downstream, may effectively dissipate flow energy during floods (Entwistle et al. 2018). Other portions, such as floodplain wetlands, may retain water and limit the effects of wildfire and droughts (Fairfax and Whittle 2020). Heterogeneity equates to patches and gradients in habitats, conditions, and resources that support stable populations and greater biotic diversity (Van Looy et al. 2019, Pelletier et al. 2020). Recent research in terrestrial ecosystems also suggests that spatial self-organization into regular vegetation patterns or patches in the landscape can allow ecosystems to evade tipping points and therefore enhance resilience (Rietkerk et al. 2021). Consequently, diverse lines of evidence indicate that greater spatial heterogeneity tends to increase river corridor absorbing capacity, but the maintenance of this heterogeneity relies on river processes such as lateral channel movement and overbank flow, as well as appropriate space in which the processes can operate. In other words, spatial heterogeneity relies on physical integrity.

Temporal heterogeneity describes variation in river corridor form and function and ecological communities over diverse timespans, from changes in floodplain vegetation and channel planform caused by the short disturbance of a single large flood (Friedman and Lee 2002), to progressive channel incision and formation of terraces because of the long-term disturbance caused by relative base-level lowering (e.g., Castillo et al. 2013). Organisms exploit the longitudinal and lateral connectivity of rivers and floodplains and temporally varying flows to access resources across river networks and floodplains over different timescales, which affects population dynamics and community structure over time (Wolter et al. 2015). Temporal heterogeneity in process and form creates and maintains spatial heterogeneity and creates a shifting habitat mosaic (Arscott et al. 2002, Stanford et al. 2005) that can promote biodiversity and absorbing capacity.

Existing frameworks categorize portions of a catchment and different types of river corridors (e.g., bedrock canyon versus lowland floodplain river versus spring-fed headwaters) in terms of geomorphic process domains (Montgomery 1999), river styles (Brierley and Fryirs 2005), river corridor morphology (Stanford and Ward 1993), flow regime (Poff 1996), freshwater and riparian communities (Aarts and Nienhuis 2003), and other criteria. These frameworks can be used to delineate spatial variations in riverine forms and functions, as well as the likely relative importance of specific characteristics of, and metrics indicating, absorbing capacity for a particular portion of a river.

## Restoring for river absorbing capacity

We briefly review current restoration approaches and measures of restoration success before presenting a conceptual framework for measuring river corridor absorbing capacity.

### Current restoration approaches

As summarized in several recent reviews and syntheses (Wohl et al. 2015b, Roni et al. 2019, Polvi et al. 2020), river restoration is interpreted quite diversely and can be used to describe river management that is focused on conflicting esthetic preferences (Kondolf et al. 2013, Zingraff-Hamed et al. 2018), channel stability (Brookes and Shields 1996), or specific management goals, such as flood control (Dadson et al. 2017, Veról et al. 2019), water quality (Viswanathan and Schirmer 2015), sustaining individual species (e.g., Null and Lund 2012), or on restoring rivers as ecosystems (Doyle and Drew 2008, Beechie et al. 2010). Although *rehabilitation* is sometimes distinguished from *restoration* (e.g., Sear 1994, Smith et al. 2014, Fryirs and Brierley 2016, Newson 2022, Fryirs 2024), we use *restoration* to encompass any management activity within or outside of the river corridor that is intended to sustain or enhance river ecosystem functionality (e.g., Chazdon et al. 2024).

River restoration at the end of the twentieth century and continuing into the twenty-first century has gradually shifted from an emphasis on engineering for stability and downstream conveyance of materials, to working with rivers as dynamic entities (Brookes 1987, Beechie et al. 2010, Palmer et al. 2014, Johnson et al. 2020, Fryirs and Brierley 2022, Russell et al. 2023). Approaches and philosophies for restoration best practices have evolved in conjunction with growing knowledge of river corridor resilience and management of river resilience (Fuller et al. 2019, Fuller and Conley 2024). Contemporary restoration approaches include notions of working with natural processes and partnering with ecosystem engineers (e.g., Brookes and Shields 1996). In such conditions, restoration might be termed assisted natural recovery, with management intervention focused on removal of infrastructure that prevented sediment transport and erosion or deposition from making the morphological adjustments necessary to restore the river. The understanding of the role of large wood, then vegetation, and subsequently other biota has led to calls for biomic restoration approaches (Johnson et al. 2020), although biomic restoration can be limited in the heavily modified landscapes of industrialized regions.

Simultaneously, new terminology has developed around ecosystem-based or functional approaches to restoring specific processes, such as natural flood management (Dadson et al. 2017, Lane 2017), nature-based solutions (WWAP 2018), process-based restoration and natural infrastructure (Ciotti et al. 2021, Norman et al. 2022, Skidmore and Wheaton 2022), and rewilding (Newson 2022). Although the terminology used to describe this shift varies between countries and between regions and changes over time (table 2), the underlying theme is the same: enhancing the absorbing capacity of river corridors so they can withstand and adapt to future disturbances while recovering more natural process, form, and function to improve health and provision of ecosystem services (Fryirs 2024). Most of these forms of river restoration rely on expansion of river process space, restored connectivity, use of natural energy in restoration, use of native materials and organisms, time for recovery trajectories to occur, and adaptive management (Ciotti et al. 2021).

**Table 2.** Commonly used descriptions of river restoration.

**Concept**	**Description**	**Sample references**
Rewilding	restoration that promotes the natural recovery of ecosystems, through (initial) active or passive removal of human influence, and introduction of keystone species	Jones and Comfort et al. 2020, Rideout et al. 2021
Renaturalization or naturalization	shifting some components of an altered ecosystems closer to a natural condition, while maintaining or enhancing existing economic and social uses of the ecosystem	Rhoads and Herricks 1996, Sparks et al. 1998, Gorczyca et al. 2018
Room for rivers, erodible river corridor, or freedom corridor	reduces flood and bank erosion risks by enhancing flood conveyance via setback of artificial levees rather than continually increasing levee height	Klijn et al. 2018
Recovery enhancement	emphasizes where recovery from anthropogenic alteration is occurring and how this can be enhanced	Fryirs et al. 2018
Process-based restoration	focuses on reestablishing normative rates and magnitudes of physical, chemical, and biological processes that sustain river ecosystems	Beechie et al. 2010, Palmer and Ruhi 2019
Natural flood management	emphasis on catchment-scale manipulation to reduce flood inundation	Lane 2017
Soft or bioengineering	focuses on green or natural infrastructure (e.g., riparian vegetation, beaver dams, engineered logjams) with respect to hazard reduction	Fernandez and Ahmed 2019

### Measuring restoration success

A difficult question to address is the extent to which restoration activity has accomplished its stated aims. This question embodies notions of the scale of restoration relative to the scale of the goals and the scale of modifications and the success of restoration projects in delivering river ecosystems with absorbing capacity.

Inventories of river restoration exist for different nations and regions (e.g., Bernhardt et al. 2005, Palmer et al. 2014, Viswanathan and Schirmer 2015, Cashman et al. 2019, Fryirs et al. 2021). Many projects report the simple metric of length of channel restored. Existing databases and inventories may have captured the structural characteristics of river corridor absorbing capacity (physical integrity, spatial heterogeneity) but failed to assess the functional resilience and viability of river corridors as ecosystems. Ideally, other measures would be included that account for rivers as three-dimensional corridors, such as area of river ecosystem restored, strength of connectivity repaired, and indicators of native biotic community response.

The appropriate scale of restoration is defined in part by connectivity of process (e.g., bedload tends to be more locally sourced, wash load reflects catchment land surface processes), whereas ecosystem restoration requires attention to life history traits and patch connectivity, with recent emphasis on connectivity of multiple smaller, diverse patches (Jacquet et al. 2022). Several observers note that restoration failures are commonly caused less by the scale of the project than by the failure to identify the key stressors causing the loss of ecological integrity or the mismatch between scale of restoration and the scale over which stressors affect the river system (Sear 1994, Bernhardt and Palmer 2011). Consequently, even after decades of river restoration, it remains unclear whether restoration is making rivers more resilient or increasing their absorbing capacity. River scientists and practitioners still have much to learn in terms of assessing river absorbing capacity and functional responses, including the appropriate scales and conditions under which resilient rivers can be realistically restored.

Closely related questions involve how much absorbing capacity can be—or, ideally, should be—restored. The question of how much can be restored or recovered can involve site-specific constraints that cannot be mitigated (e.g., alterations to flow regime that cannot be reversed or exotic species that cannot be eliminated), as well as trade-offs between restoration actions that increase absorbing capacity and loss of features perceived as beneficial by society (e.g., increasing physical integrity, spatial heterogeneity, and lateral connectivity by removing artificial levees versus reducing agriculture or infrastructure in the floodplain). The question of how much absorbing capacity ideally should (or could) be restored can be assessed by measuring desired outcomes of restoration (e.g., water quality, fish biomass and biodiversity) and adapting restoration actions as possible to achieve these desired outcomes. In a world with rapidly changing disturbance regimes, it seems wise to err on the side of caution and exceed desired outcomes where possible.

### A conceptual framework for measuring river corridor absorbing capacity

Given the paucity of pre- or postproject monitoring data from past river restoration projects (Palmer et al. 2007), the detection of changing absorbing capacity has been difficult. There is a fundamental need to define indices of river absorbing capacity that capture both the different components of the river ecosystem (e.g., trophic levels, channel adjustment capacity) and the variable policy-driven requirements of restoration (Parsons and Thoms 2018). Many such measures explicitly recognize the need for metrics of absorbing capacity to be nested within the wider catchment (e.g., Beechie et al. 2023). For example, ecological concepts, such as dispersal capacity, require an understanding of the expected connectivity of a restored reach measured relative to population sources, density of species in these sources, and quality of habitat and food availability for colonizing organisms in the restored reach (Jaquet et al. 2022). Such an approach requires consideration of areas well outside the target restoration reach.

The absorbing capacity of restored river corridors must also include the inherent risks to restoration goals that are typically beyond the scope of a restoration project. Examples include the presence of invasive species in the wider catchment and unresolved catchment pressures, such as water quality or flow regulation, that might limit the capacity for the system to adjust or that may prompt new undesirable system states.

Measurements of absorbing capacity will need to vary. Fast (e.g., extreme flood or drought) versus slow (climate change) disturbance may require different indices of absorbing capacity. Long-term monitoring is possible for river corridors through sedimentary archives stored in floodplain deposits and paleochannels (Sear and Arnell 2006, Brown et al. 2018) that record gradual changes in the river corridor ecosystem over annual to millennial timescales. Similarly, historic mapping and remote sensing can be used to detect temporal changes in channel pattern and rates of adjustment over decades to centuries (Petts 1989). Vegetation communities and water table depths in floodplain sediments are also detectable using archives of remote sensing and ever-advancing sensor technology (Lenton et al. 2022). The response of a river ecosystem to short-term disturbances can also be detected using remotely sensed data and sensors that are collected at shorter time intervals.

**Figure 3.**
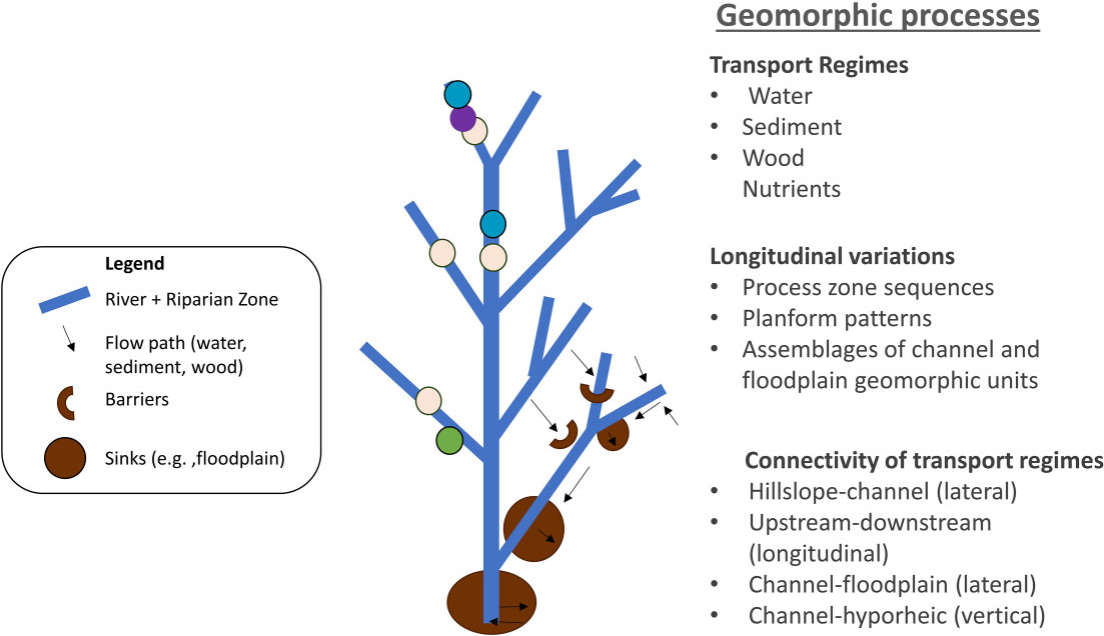
Schematic illustration of geomorphic processes influencing river corridor characteristics that could be measured in an integrated approach to measuring absorbing capacity of a river corridor (see the supplemental material for a more complete list of potential characteristics and metrics). Transport regimes and connectivity are described in the text. Longitudinal variations refers to downstream variation in the dominant geomorphic process (e.g., debris flow versus snowmelt flood versus rainfall flood), channel planform (e.g., meandering versus braided), and assemblages of channel units such as steps and pools or pools and riffles or floodplain units such as backswamps and levees.

No single measurement or metric of absorbing capacity will be sufficient for all scenarios of disturbance and response in river corridors. Metrics need to be tailored to the system under consideration. Depending on the degree of human-induced change, including climate change, metrics of absorbing capacity might be developed relative to an expected reference condition (Brierley et al. 2010, Fryirs 2015). Alternatively, metrics of river corridor absorbing capacity might look toward novel ecosystems that can provide a range of ecosystem services and be sustainable under existing conditions (Francis 2014, Thorel et al. 2018). The metrics used must also be sensitive enough to detect and quantify absorbing capacity for the full river corridor and consider processes and pressures operating outside the target work areas. In figure 3, we provide an overview of river corridor characteristics that could be measured in an integrated approach to measuring river absorbing capacity.

## Toward improved river corridor absorbing capacity

We suggest that the key next steps in restoring river absorbing capacity involve considering what scale of restoration is required to deliver river corridor absorbing capacity for different riverscapes; understanding sociopolitical elements of river corridor absorbing capacity and restoration; and defining relevant measures of river corridor absorbing capacity that are fit for purpose and can be used to improve forecasting capability for possible future states.

### Considering scale

Restoring and maintaining river corridor absorbing capacity must include restoring catchment-scale fluxes that drive the processes that support absorbing capacity (Sear 1994, Wheaton et al. 2018, Angelopoulos et al. 2017, Rocha et al. 2022). Given the small impact, high cost, and slow rate of restoration relative to the scale of modified river ecosystems, the current approaches to restoration need to change. Two broad changes in restoration actions are required: first, increased adoption of working with natural processes to deliver restoration (Beechie et al. 2010, Palmer and Ruhi 2019, Fryirs and Brierley 2022). This approach includes, among other things, rethinking the perceived impacts of large floods. Currently, the responses to large floods are focused on repairing damage and supporting affected communities, but expenditure on returning to a preflood river configuration fails to recognize the costs to river corridor absorbing capacity. Working with organisms that act as ecosystem engineers (e.g., large wood, vegetation, beaver, benthic-spawning fish) represents a cheaper alternative to physical, energy-intensive channel design work that typically includes the use of heavy construction equipment (e.g., Pollock et al. 2014).

The second required change is to select reach-scale restoration sites more strategically within a catchment context to maximize benefits (Agnew and Fryirs 2022). Restoration priorities are typically based on site accessibility or stakeholder support, rather than optimizing potential benefits. Progress is being made with strategic restoration planning and prioritization (e.g., the Danube River catchment, Nachtnebel 2000, and the Sacramento–San Joaquin River system in California, Lacan and Resh 2016), but to date, this has tended to be narrowly focused on achieving discipline-bound targets or policy objectives. Site selection and restoration measures must consider the local requirements for the target species but should be informed by catchment assessments of habitat availability, accessibility, and quality (Van Looy et al. 2019, Pelletier et al. 2020).

Fundamentally, the challenge in considering scale lies in determining and quantifying absorbing capacity, which is a function of large-scale drivers, such as climate and landscape setting, past and prevailing management regimes, and whether these drivers are manifesting as expected at the site, reach, river corridor, and river network scales without exceeding critical tipping points.

### Understanding sociopolitical elements

Systems perceived as natural do not always exist in a state unmodified by humans, restored natural processes will operate in modified spaces, and climate continues to change (Brown et al. 2018). Restoration at scales relevant to long-term resilience in the face of climate change and the ongoing intensification of anthropogenic modification demands a transformation in current restoration practice. Key barriers to upscaling restoration (as outlined in the UN Decade of Ecosystem Restoration) lie in the political will, governance structures, and funding to make transformational change, and the mosaic of land ownership that currently disconnects restoration actions and confines them to short actions typically focused on river channels (Russell et al. 2023, UNEP 2024).

In the context of land ownership, there is a need to incentivize riparian landowners and communities to deliver ecosystem services and build natural capital through coproduced restoration plans and activities. Ultimately, restoration is a human-mediated process, and building communities of restoration practice along rivers is necessary to overcome fragmented land ownership, the typically unidirectional transfer of knowledge, and top-down driven implementation of small-scale river restoration projects (Rogers 2006, Roux et al. 2006).

There is a fundamental need to build a diverse and inclusive river restoration community that respects, acknowledges, and uses diverse societal and traditional knowledge, including that of First Nations peoples (Wilcock et al. 2013, Ashmore 2015, Fox et al. 2017, Brierley 2019, Koppes 2022). Examples include restoration projects undertaken by indigenous peoples on tribal lands in North America, with goals and implementation undertaken by the community (Fox et al. 2017) and efforts in Aotearoa New Zealand, where the rights of the river are central (Brierley 2019).

### Defining relevant measures

To adequately assess and forecast river corridor absorbing capacity requires much better understanding of cross-scalar spatial and temporal dynamics in river corridor ecosystems across the full spectrum of river diversity. Although monitoring data and numerical models can be used to understand river corridor evolutionary trajectories (Brierley and Fryirs 2016), these efforts remain hampered by limited incorporation of biophysical processes; simplified physical representation of the river corridor; limited ability to quantify feedback loops, nonlinear and emergent behavior, and alternative states (Livers et al. 2018, Wohl 2019); and continuing legacy effects (James 2013, Wohl 2015).

In river restoration, the use of paleoenvironmental data has extended the timescales of monitoring for some variables (e.g., invertebrate diversity, fish abundance, flood frequency, discharge, morphology). This enables identification of critical transitions and tipping points in relation to different disturbance pressures (e.g., Sear and Arnell 2006, Croke et al. 2016, 2021, Hesse et al. 2018a, 2018b). These then allow identification of trajectories of change and their drivers, within which the current river ecosystem is situated. However, it is rare that such analyses are used to inform river corridor restoration practice (Croke et al. 2021); undertake forecasting exercises to identify possible future states; guide understandings of the likelihood of achieving river corridor resilience; and prioritize where on-the-ground restoration actions will have best return on investment (Trofimov and Phillips 1992, Wilcock and Iverson 2003, Fryirs et al. 2018, Newson 2022).

Modeling plausible states within which restoration outcomes sit emphasizes that multiple states of restored rivers could exist as an outcome of restoration activity, while recognizing boundaries as to what the restored system could attain (Sear et al. 2008). Metrics of absorbing capacity can be identified, such as strength and number of negative feedback loops in the system required to dampen the impact of a disturbance (Scheffer et al. 2015), the self-organized structure of the system (e.g., McCluney et al. 2014), and ecological communities (Van Looy et al. 2019). However, complex river ecosystems with multiple feedback loops and exposure to varied disturbances across the system mean that predicting restoration outcomes remains uncertain (Darby and Sear 2008). In similar systems (e.g., lake restoration; Stow et al. 2020), adaptive management based on monitoring and adjustments has embraced uncertainty and adapted restoration practices to attain desired outcomes or, where restorative actions fail, these have been modified or stopped. In river restoration, monitoring has been consistently short term (years), if done at all, nonstandardized, and typically limited in scope. Monitoring is, however, a key attribute providing information (evidence) to make changes, and to promote learning and contagion (Hunter and Brown 2012), whereby other communities see the possibility and benefits of restoration.

**Figure 4.**
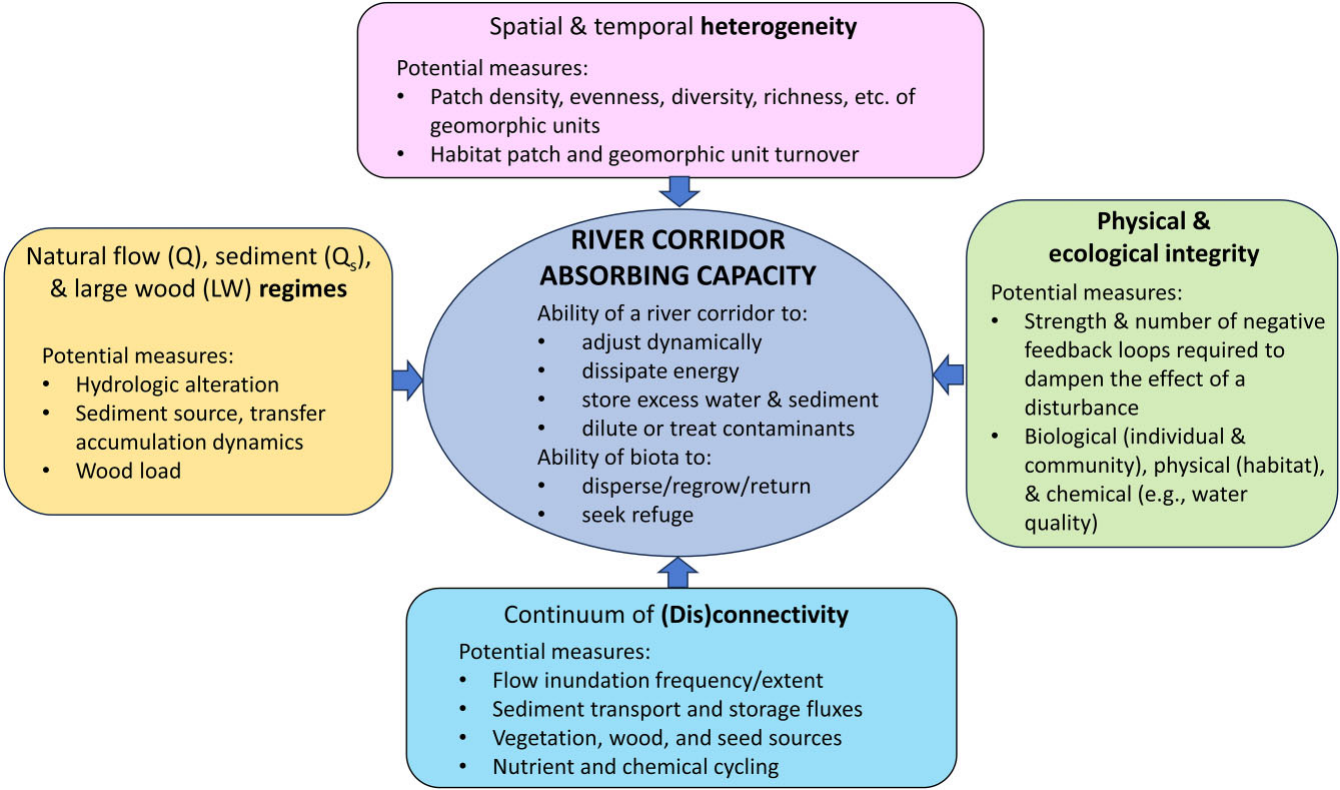
An integrative illustration of potential metrics of river absorbing capacity associated with the processes and features that contribute to absorbing capacity. (See the supplemental material for additional suggestions).

Resilience as a general concept has been critiqued as largely theoretical and lacking clearly defined metrics or indices (Scheffer et al. 2015). This remains a challenge in river management because of the contingent and highly varied conditions under which rivers operate and the wide range of diversity of rivers that occur in the landscape. We do not consider it practical or appropriate in this article to define the metrics of resilience or absorbing capacity that will apply to all river restoration scenarios because of the diversity of both river catchments and corridors and the constraints that apply to individual sites at which restoration is undertaken. However, we propose a general flowchart and suggested approaches for establishing metrics of absorbing capacity for individual restoration projects (figure 4, supplemental material). The supplemental material lists catchment- and reach-scale parameters that should be considered when measuring absorbing capacity, as well as the specific features of these parameters (e.g., magnitude and frequency for flow or sediment regimes), quantitative metrics where available, and appropriate references. A key point in characterizing any of these parameters and metrics is that the relative values are likely to be most important, either in the context of variations along a river or within a river network or in the context of natural versus human-altered conditions.

## Summary and future perspectives

Resilience and absorbing capacity emerge from the nonlinear interactions of river corridor structures and functions across various temporal and spatial scales. The transformations that emerge from cross-scalar dynamics are poorly understood, both within river systems and conceptually within a resilience framework. Humans have disrupted many river dynamics through the simplification of river corridor structure and dynamics, leading to less resilient systems that could benefit from restoration activities.

River corridors continue to provide vital ecosystem services, despite centuries of human alterations that have simplified and homogenized them and reduced their capacity to absorb natural and human disturbances. River restoration that situates local management actions within a catchment context and protects or restores the ability of the river to physically and ecologically adjust presents opportunities for increasing the absorbing capacity of the river corridor. This restoration occurs in a societal context that includes infrastructure and conflicting human perceptions and desires (Hawley 2018). River restoration also occurs in an extraordinarily complex physicoecological context that river scientists remain challenged to understand and predict, especially in a time of changing climate. By focusing on defining measures of absorbing capacity that are fit for purpose for the river corridor under investigation (figure 4), understanding the scale of restoration required to enhance absorbing capacity, and explicitly incorporating sociopolitical elements, we believe that river restoration can improve the resilience of river corridors, better preparing human societies for an uncertain future.

## Supplementary Material

biae090_Supplemental_File

## Author Biography

Ellen Wohl (ellen.wohl@colostate.edu) is affiliated with the Department of Geosciences at Colorado State University, in Fort Collins, Colorado, in the United States. Kirstie Fryirs is affiliated with the School of Natural Sciences at Macquarie University, in North Ryde, New South Wales, in Australia. Robert C. Grabowski is affiliated with the Centre for Water, Environment, and Development at Cranfield University, in Cranfield, England, in the United Kingdom. Ryan R. Morrison is affiliated with the Department of Civil and Environmental Engineering at Colorado State University, in Fort Collins, Colorado, in the United States. David Sear is affiliated with the Department of Geography and Environmental Science at the University of Southampton, in Southampton, England, in the United Kingdom.
