# Supplementary material for: Enhancing the natural absorbing capacity of rivers to restore their resilience
Source: Bioscience. 2024 Sep 19;74(11):782–96. doi: 10.1093/biosci/biae090 (PMC11565886; doi:10.1093/biosci/biae090)
Supplement: biae090_Supplemental_File [file biae090_supplemental_file.docx]

**Supplemental Material: A general flow-chart and suggested approaches for establishing metrics of river corridor absorbing capacity**

Phase I. Characterize Natural Context

- Catchment-scale parameters
  - flow regime
    - magnitude, frequency, duration, timing, connectivity and rate of change of flows (Poff et al., 1997)
  - sediment regime
    - magnitude, frequency, duration, timing, connectivity and rate of sediment inputs & fluxes (Walling & Collins, 2008; Wohl et al., 2015)
  - large wood regime
    - magnitude, frequency, duration, timing, rate, and mode of wood movement (Wohl et al., 2019)
  - nutrient budget (Sferatorre et al 2005)
  - form-process and erosion-deposition patterns
    - process zone patterns (source-transfer-accumulation zones)
    - planform patterns (sequence of river types)
  - assemblages of channel and floodplain geomorphic units
  - (dis)connectivity (water, sediment, large wood, & nutrients moving downslope/downstream, organisms moving in all directions)
  - presence, spatial distribution, & sustainability of ecosystem engineers and seed banks and their connectivity to the river network
  - biodiversity metrics, likely most effectively applied at the sub-catchment scale, with details of scale dependent on dispersal abilities of organism(s)
- Reach-scale parameters
  - flow regime (as for the catchment)
  - sediment regime (as for the catchment; Fuller et al., 2003)
  - large wood budget & process domain (Benda & Sias, 2003; Wohl et al., 2019)
  - nutrient budget (Brunel & Astin, 2000)
  - form-process and erosion-deposition patterns (as for the catchment)
  - 3D connectivity
    - lateral: floodplain inundation frequency, spatial extent, duration & associated movement of sediment, solutes, large wood, seedbanks & organisms; multiple measures depending on material being considered (e.g., Wohl et al., 2017)
    - vertical: hyporheic exchange flows magnitude, spatial extent, duration & associated movement of solutes & organisms (Harvey et al., 2019)
    - longitudinal: downstream variation in flow conveyance, sediment transport, large wood transport, nutrient uptake, organism movement, propagule sources; multiple measures depending on material being considered (e.g., Wohl, 2017)
  - biodiversity
    - biodiversity metrics (greater values of metrics = greater resilience) (Santini et al., 2017)
  - functional diversity
    - metrics of functional richness, evenness, divergence, & dispersion (greater functional diversity = greater resilience) (Kuebbing et al., 2018)
  - spatial heterogeneity
    - heterogeneity metrics such as patch density, evenness, diversity, and richness of biogeophysical units (greater heterogeneity = greater resilience) (Iskin & Wohl, 2023)
    - turnover of geomorphic units and habitat patches
  - temporal heterogeneity
    - characterize magnitude of change through time in the absence of human alterations & relevant timespans (e.g., regular shift between braided & meandering, Friedman & Lee, 2002)

Phase II. Identify Degree of Human Modification

- Catchment-scale parameters
  - altered flow regime
    - indicators of hydrologic alteration (Richter et al 1996) (lesser alteration = greater resilience)
    - ecological limits of hydrologic alteration (Poff et al., 2010) (proximity to limits = lesser resilience)
  - altered sediment regime
    - indirect (e.g., comparison of historical vs contemporary indicators of sediment dynamics, such as channel cross-sectional geometry, bed substrate, or channel planform complexity; Wohl et al., 2015)
  - altered large wood regime
    - indirect (e.g., comparison of historical vs contemporary indicators of potential large wood recruitment, storage, & transport)
  - altered nutrient regime
    - indirect (e.g., comparison of altered land cover & land use as an effect on nutrient inputs, or comparison of altered river network configuration & connectivity as an effect on nutrient uptake)
  - altered form-process and erosion-deposition patterns
    - change in process zone sequences, planform patterns, assemblages of channel and floodplain geomorphic units
  - connectivity
    - identify sources of change in connectivity (e.g., transportation corridors (Blanton & Marcus, 2009), artificial levees (Knox et al., 2022), or flow regulation (Ward & Stanford, 1995) for lateral connectivity, or dams for longitudinal connectivity (Grill et al 2019; Jumani et al., 2020))
  - physical integrity
    - proportion of total river network or location of individual segments of river network that have reduced ability to respond to changes in inputs because of human constraints (e.g., bank stabilization, artificial levees, flow regulation, channelization)
  - ecological integrity (greater integrity = greater resilience)
    - biotic integrity metrics (e.g., Karr, 1981; Harris & Silveira, 1999)
  - spatial heterogeneity
    - heterogeneity metrics (homogenization relative to natural conditions = lesser resilience)
  - temporal heterogeneity
    - characterize magnitude & timespan of changes likely attributable to human alterations (e.g., Trimble, 2013)
- Reach-scale parameters
  - altered flow regime (as for the catchment)
  - altered sediment budget
    - capacity supply ratio (Soar & Thorne, 2001), S* (Schmidt & Wilcock, 2008), τ* (Grant et al., 2003)
  - altered wood budget & process domain
    - indicators of altered wood dynamics: reduced recruitment (change in catchment & reach land cover), transport (change in wood supply & flow), & storage (reduced trapping capacity of reach; Scott & Wohl, 2018) of large wood
    - indicators of altered secondary effects from wood dynamics (changes in pool volume, bed substrate grain size & mobility, aquatic & riparian habitat abundance & diversity, & aquatic & riparian biota abundance & diversity – all as associated with large wood; Wohl et al., 2019)
  - altered nutrient budgets
    - indirect (comparison of likely historical vs contemporary nutrient budget; e.g., the nitrate time bomb (Wang et al., 2013))
  - altered form-process and erosion-deposition patterns
    - directional changes through time in proxy indicators such as channel cross-sectional geometry, number and successional stages of bars & islands, channel substrate grain-size distribution & particle stability, channel planform, floodplain alluvial volume & turnover time, or spatial heterogeneity of species & plant ages (Wohl et al., 2015)
  - 3D connectivity
    - as for catchment scale, identify sources of changes in connectivity
  - physical integrity
    - currently available accommodation space relative to natural accommodation space (higher ratio = greater resilience)
    - strength & number of feedback loops required to dampen the effect of a disturbance (greater strength & numbers = lesser resilience)
  - ecological integrity (as for catchment scale)
  - functional diversity
    - diversity metrics (lower values relative to natural conditions = lesser resilience)
  - spatial heterogeneity
    - heterogeneity metrics (homogenization relative to natural conditions = lesser resilience)
  - temporal heterogeneity (as for catchment scale)

Phase III. Management of Resilience

- identify most/least resilient subcatchments within a larger catchment or reaches along a river
- identify subcatchments or reaches in which management can potentially increase resilience
- manage the (dis)connectivity prior to physical reach-scale restoration to support resilience targets (Sear, 1994)

**References**

Benda LE, Sias JC. 2003. A quantitative framework for evaluating the mass balance of in-stream organic debris. Forest Ecology and Management 172: 1-16. <https://doi.org/10.1016/S0378-1127(01)00576-X>

Blanton P, WA. 2009. Railroads, roads and lateral disconnection in the river landscapes of the continental United States. Geomorphology 112: 212-227. <https://doi.org/10.1016/j.geomorph.2009.06.008>

Brunet RC, Astin KB. 2000. A 12-month sediment and nutrient budget in a floodplain reach of the River Adour, southwest France. Regulated Rivers: Research and Management 16: 267-277. <https://doi.org/10.1002/(SICI)1099-1646(200005/06)16:3%3C267::AID-RRR584%3E3.0.CO;2-4>

Friedman JM, Lee VJ. 2002. Extreme floods, channel change, and riparian forests along ephemeral streams. Ecological Monographs 72: 409-425. [https://doi.org/10.1890/0012-9615(2002)072[0409:EFCCAR]2.0.CO;2](https://doi.org/10.1890/0012-9615(2002)072%5b0409:EFCCAR%5d2.0.CO;2)

Fuller IC, Large ARG, Charlton ME, Heritage GL, Milan DJ. 2003. Reach-scale sediment transfers: an evaluation of two morphological budgeting approaches. Earth Surface Processes and Landforms 28: 889-903. <https://doi.org/10.1002/esp.1011>

Grant GE, Schmidt JC, Lewis SL. 2003. A geological framework for interpreting downstream effects of dams on rivers. In, A Peculiar River: Geology, Geomorphology, and Hydrology of the Deschutes River, Oregon, JE O’Connor, GE Grant, eds. American Geophysical Union, Washington, DC, 203-219.

Grill G, Lehner B, Thieme M, Geenen B, Tickner D, Antonelli F, Babu S, Borrelli P, Cheng L, Crochetiere H, et al. 2019. Mapping the world’s free-flowing rivers. Nature 569: 215-221. <https://doi.org/10.1038/s41586-019-1111-9>

Harris JH, Silveira R. 1999. Large-scale assessments of river health using an Index of Biotic Integrity with low-diversity fish communities. Freshwater Biology 41: 235-252. <https://doi.org/10.1046/j.1365-2427.1999.00428.x>

Harvey J, Gomez-Velez J, Schmadel N, Scott D, Boyer E, Alexander R, Eng K, Golden H, Kettner A, Konrad C, et al. 2019. How hydrologic connectivity regulates water quality in river corridors. Journal American Water Resources Association 55: 369-381. <https://doi.org/10.1111/1752-1688.12691>

Iskin EP, Wohl E. 2023. Beyond the case study: Characterizing natural floodplain heterogeneity in the United States. Water Resources Research 59: e2023WR035162. <https://doi.org/10.1029/2023WR035162>

Jumani S, Deitch MJ, Kaplan D, Anderson EP, Krishnaswamy J, Lecours V, Whiles MR. 2020. River fragmentation and flow alteration metrics: a review of methods and directions for future research. Environmental Research Letters 15: 123009. <https://iopscience.iop.org/article/10.1088/1748-9326/abcb37/meta#:~:text=DOI%2010.1088/1748%2D9326/abcb37>

Karr JR. 1981. Assessment of biotic integrity using fish communities. Fisheries 6: 21-27. <https://doi.org/10.1577/1548-8446(1981)006%3C0021:AOBIUF%3E2.0.CO;2>

Knox RL, Morrison RR, Wohl EE. 2022. A river ran through it: Floodplains as America’s newest relic landform. Science Advances 8: eabo1082. <https://doi.org/10.1126/sciadv.abo1082>

Kuebbing SE, Maynard DS, Bradford MA. 2018. Linking functional diversity and ecosystem processes: A framework for using functional diversity metrics to predict the ecosystem impact of functionally unique species. Journal of Ecology 106: 687-698. <https://doi.org/10.1111/1365-2745.12835>

Poff NL, Richter BD, Arthington AH, Bunn SE, Naiman RJ, Kendy E, Acreman M, Apse C, Bledsoe BP, Freeman MC, et al. 2010. The ecological limits of hydrologic alteration (ELOHA): A new framework for developing regional environmental flow standards. Freshwater Biology 55: 147-170. <https://doi.org/10.1111/j.1365-2427.2009.02204.x>

Richter BD, Baumgartner JV, Powell J, Braun DP. 1996. A method for assessing hydrologic alteration within ecosystems. Conservation Biology 10: 1163-1174. <https://doi.org/10.1046/j.1523-1739.1996.10041163.x>

Santini L, Belmaker J, Costello MJ, Pereira HM, Rossberg AG, Schipper AM, Ceausu S, Dornelas M, Hilbers JP, Hortal J, et al. 2017. Assessing the suitability of diversity metrics to detect biodiversity change. Biological Conservation 213B: 341-350. <https://doi.org/10.1016/j.biocon.2016.08.024>

Sferatorre A, Billen G, Garnier J, Thery S. 2005. Modeling nutrient (N, P, Si) budget in the Seine watershed: Application of the Riverstrahler model using data from local to global scale resolution. Global Biogeochemical Cycles 19. <https://doi.org/10.1029/2005GB002496>

Soar PJ, Thorne CR. 2001. Channel restoration design for meandering rivers. ERDC/CHL Report CR-01-1. US Army Corps of Engineers, Vicksburg, MS.

Schmidt JC, Wilcock PR. 2008. Metrics for assessing the downstream effects of dams. Water Resources Research 44. <https://doi.org/10.1029/2006WR005092>

Scott DN, Wohl EE. 2018. Natural and anthropogenic controls on wood loads in river corridors of the Rocky, Cascade, and Olympic Mountains, USA. Water Resources Research 54: 7893-7909. <https://doi.org/10.1029/2018WR022754>

Trimble SW. 2013. Historical Agriculture and Soil Erosion in the Upper Mississippi Valley Hill Country. CRC Press, Boca Raton, FL. <https://doi.org/10.1201/b13039>

Walling DE, Collins AL. 2008. The catchment sediment budget as a management tool. Environmental Science & Policy 11: 136-143. <https://doi.org/10.1016/j.envsci.2007.10.004>

Wang L, Butcher AS, Stuart ME, Gooddy DC, Bloomfield JP. 2013. The nitrate time bomb: a numerical way to investigate nitrate storage and lag time in the unsaturated zone. Environmental Geochemistry and Health 35: 667-681. <https://doi.org/10.1007/s10653-013-9550-y>

Ward JV, Stanford JA. 1995. Ecological connectivity in alluvial river ecosystems and its disruption by flow regulation. Regulated Rivers: Research and Management 11: 105-119. <https://doi.org/10.1002/rrr.3450110109>

Wohl E. 2017. Connectivity in rivers. Progress in Physical Geography 41: 345-362. <https://doi.org/10.1177/0309133317714972>
